# From Fetal Diagnosis to Staged Percutaneous Palliation: A Case of Double Right-Valve Dysplasia

**DOI:** 10.1016/j.jaccas.2025.105852

**Published:** 2025-10-30

**Authors:** Francesca Bonanni, Adalgisa Cordisco, Giovanni Battista Calabri, Chiara Di Filippo, Giuseppe Santoro, Iacopo Olivotto, Silvia Favilli

**Affiliations:** a Meyer Children's Hospital IRCCS, Florence, Italy; b Health Science Interdisciplinary Center, Scuola Superiore Sant’Anna, Pisa, Italy; c Division of Prenatal Diagnosis, Azienda USL Toscana Centro, San Giovanni di Dio Hospital, Florence, Italy; d Fondazione Toscana Gabriele Monasterio, Pisa, Italy; e Department of Experimental and Clinical Medicine, University of Florence, Florence, Italy

**Keywords:** congenital heart defect, hemodynamics, imaging, pregnancy, tricuspid valve

## Abstract

**Background:**

Tricuspid valve dysplasia (TVD) and pulmonary valve dysplasia (PVD) are rare congenital anomalies that challenge prenatal counseling and postnatal care. When feasible, biventricular repair is the highest priority choice.

**Case Summary:**

We report a prenatal diagnosis of non-Ebstein TVD with severe regurgitation, hypoplastic right ventricle (RV), and PVD with an intact ventricular septum. Prenatal counseling illustrated the potential ventricular repair plan. During the postnatal period, pulmonary valvuloplasty was performed, followed by stenting of the ductus arteriosus. The patient demonstrated clinical improvement, a progressive increase in RV size, and satisfactory pulmonary flow over time.

**Discussion:**

This case highlights the significance of early intervention in facilitating RV growth and maximizing biventricular circulation. The literature describes staged catheter-based approaches as therapies that can avoid univentricular palliation.

**Take-Home Messages:**

Prenatal diagnosis of TVD with PVD enables timely planning and early biventricular repair, promising better long-term outcomes. A staged, personalized approach can promote RV growth.

## Visual Summary

**Figure:**
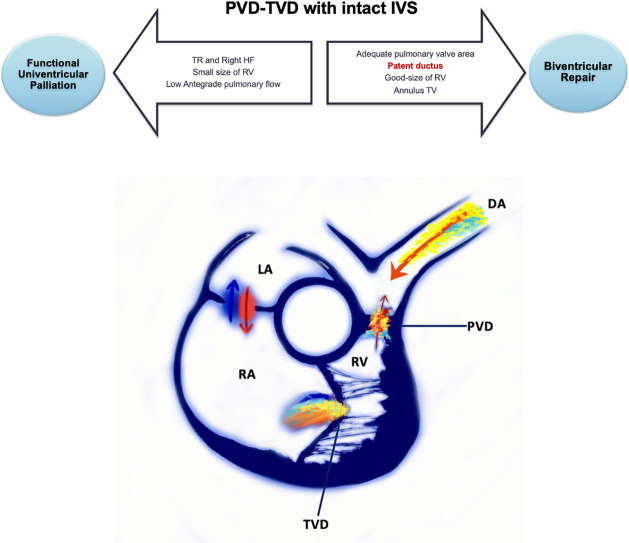

**Visual Summary:**
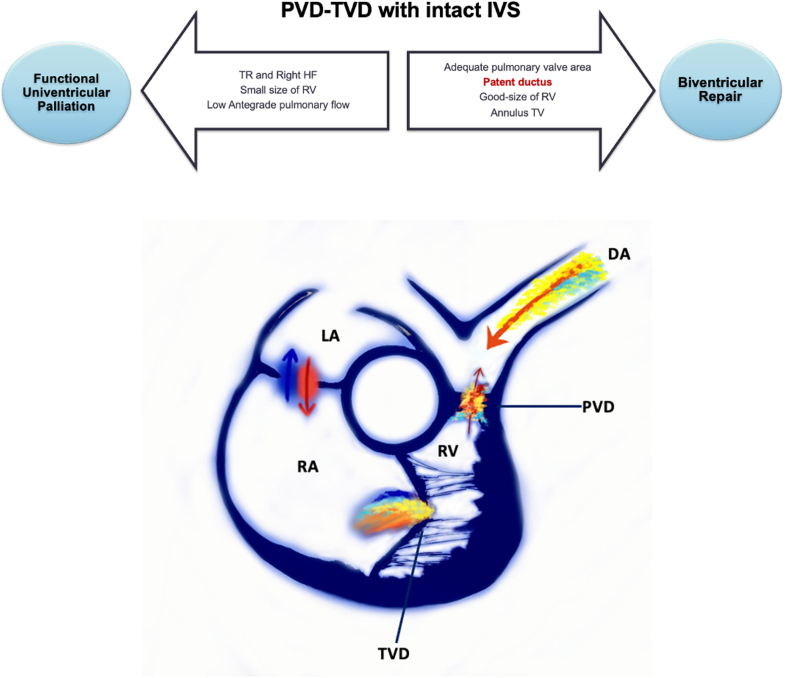
Pathway for Management Decisions in TVD and PVD With an Intact Interventricular Septum Patients presenting with severe tricuspid regurgitation, right heart failure, a small RV, and low anterograde pulmonary flow are directed toward functional univentricular palliation. Conversely, when the pulmonary valve area is adequate, a patent ductus arteriosus is maintained, and good RV and tricuspid valve dimensions are present, a staged strategy aiming for biventricular repair is feasible. The lower panel illustrates Doppler flow profiles after percutaneous pulmonary valvuloplasty and ductal stenting, promoting RV remodeling and adequate pulmonary circulation. DA = ductus arteriosus; HF = heart failure; IVS = interventricular septum; LA = left atrium; PVD = pulmonary valve dysplasia; RA = right atrium; RV = right ventricle; TR = tricuspid regurgitation; TV = tricuspid valve; TVD = tricuspid valve dysplasia.

## History of Presentation

**Figure 1:**
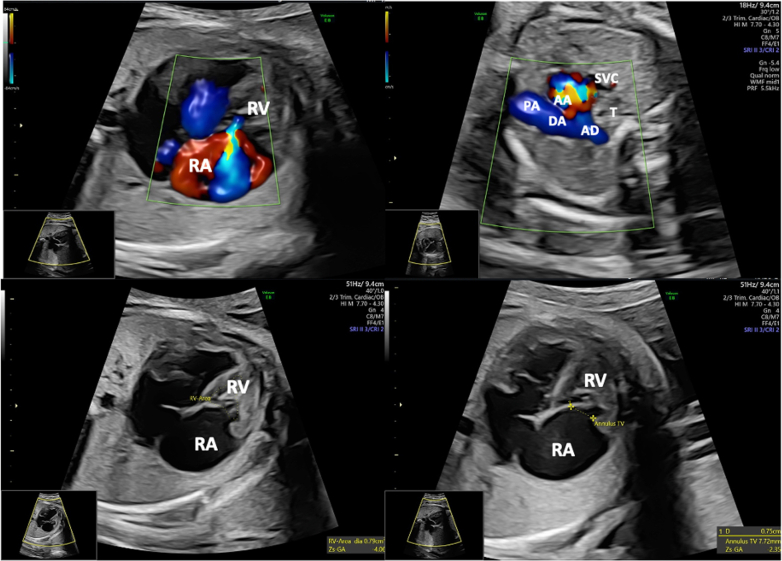
Fetal Echocardiographic Images at 32 Weeks of Gestation Showing Features of Tricuspid Valve Dysplasia and Pulmonary Valve Dysplasia With Intact Interventricular Septum (Top Left) Color Doppler 4-chamber imaging reveals severe tricuspid regurgitation with retrograde flow into the right atrium. (Top Right) Three-vessels and trachea view showing reversed blood flow from the aortic arch to the pulmonary artery. (Bottom Left) Measurement of the hypoplastic bipartite right ventricle area. (Bottom Right) Measurement of the tricuspid valve annulus, with a *z*-score of −2.41, indicating borderline right ventricular development. AA = aortic arch; AD = descending aorta; DA = ductus arteriosus; PA = pulmonary artery; RA = right atrium; RV = right ventricle; SVC = superior vena cava; T = trachea.

We present a case of prenatal diagnosis of non-Ebstein tricuspid valve dysplasia (TVD) with severe regurgitation, hypoplastic bipartite right ventricle (RV), and pulmonary valve dysplasia (PVD) with an intact ventricular septum. A routine fetal cardiac ultrasound was conducted to assess the risk of fetal hydrops. At 32 weeks, the tricuspid valve annulus *z*-score was −2.41, and retrograde perfusion of the pulmonary branches occurred via the ductus arteriosus (DA) (Figure 1).

**Take-Home Messages**

• Fetal ultrasound allowed prenatal diagnosis and timely planning for the management of complex congenital heart disease.
• Pathophysiology should guide the treatment of these patients, considering the risks and benefits of any therapeutic intervention.

## Past Medical History

The mother was primiparous, primigravida, and had negative maternal serologies. Both parents had a negative cardiovascular history and were in good health.

## Differential Diagnosis

The differential diagnosis included Ebstein anomaly, pulmonary atresia with intact ventricular septum, unbalanced atrioventricular septal defect, and Uhl anomaly. Ebstein anomaly was excluded because the septal leaflet of the tricuspid valve showed normal insertion without atrialization of the RV. Pulmonary atresia was ruled out by the presence of antegrade flow across a severely dysplastic but patent pulmonary valve. An unbalanced atrioventricular septal defect was excluded given the absence of atrioventricular valve malalignment and the presence of concordant atrioventricular connections. Finally, Uhl anomaly was considered unlikely owing to preserved myocardial thickness despite RV hypoplasia.

## Investigations

Delivery was planned at a cardiac surgery center, and the child was born at 39 weeks of gestation. At birth, he presented with mild cyanosis, rhythmic heart sounds with a systolic murmur, normal lung sounds, and normal abdominal examination results. Peripheral pulses were palpable and symmetrical. His birth weight was 3.98 kg, with height 55 cm, body mass index 13.16, and body surface area 0.24 m^2^. Oxygen saturation was 84%.

Electrocardiography showed sinus tachycardia and signs of right atrial enlargement. Abdominal ultrasound and metabolic disease screening tests returned normal results. The echocardiography revealed situs solitus and levocardia, normal atrioventricular and ventriculoarterial connections, and normal size and function of the left ventricle. It showed a hypoplastic bipartite RV, dilated right atrium, non-Ebstein TVD with moderate insufficiency and mild stenosis, and PVD with severe stenosis and an intact ventricular septum.

## Management

Subsequent cardiac catheterization showed minimal anterograde flow across the pulmonary valve, confirming severe tricuspid valve regurgitation and no RV-dependent coronary artery flow. Progressive pulmonary valve dilatation with a balloon of increasing diameter (balloon/pulmonary valve annulus ratio up to 1.3) improved hemodynamics. The procedure was well tolerated, with no complications. Over the following days, there was a reduction in tricuspid valve regurgitation and the appearance of a left-to-right shunt across the interatrial septum.

However, despite beta-blocker therapy, discontinuing PGE1 infusion led to significant oxygen desaturation, confirming persistent ductal dependency of pulmonary blood flow. Thus, PGE1 therapy was restarted, and the patient underwent stenting of the DA using a 3 × 18 mm stent dilated to 3.3 mm. This treatment allowed discontinuation of PGE1 as well as inotropic and respiratory support.

**Figure 2:**
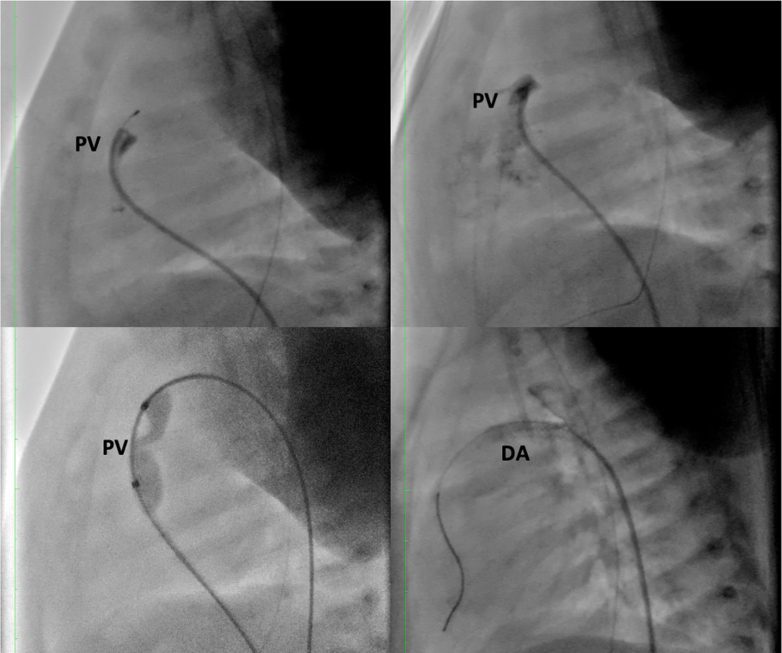
Fluoroscopic Images During Staged Percutaneous Palliation (Top Panels) Angiography of the right ventricle showing critical pulmonary valve stenosis with minimal anterograde flow; guidewire crossing the pulmonary valve. (Bottom Left) Balloon pulmonary valvuloplasty using a balloon-to-annulus ratio of 1.3. (Bottom Right) Ductal stenting with a 3.0 × 18 mm stent deployed in the ductus arteriosus to maintain adequate pulmonary blood flow. DA = ductus arteriosus; PV = pulmonary valve.

**Figure 3:**
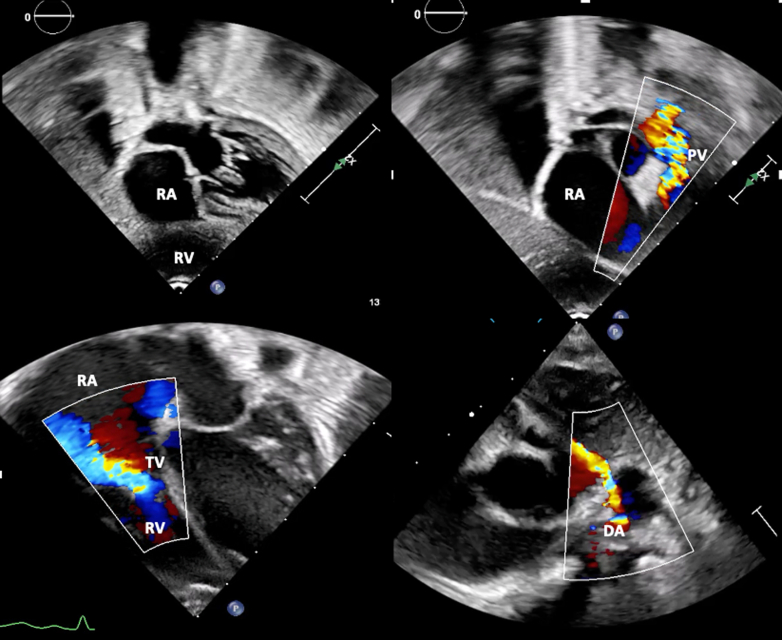
Transthoracic Echocardiography at the Time of Discharge (Top Left) Subcostal 4-chamber view shows a hypoplastic bipartite right ventricle and a dilated right atrium. (Top Right) Color Doppler imaging across the pulmonary valve demonstrates severe stenosis with minimal antegrade flow. (Bottom Left) Color Doppler assessment of the tricuspid valve shows severe to moderate regurgitation. (Bottom Right) Color Doppler of the ductus arteriosus demonstrating shunting. TV = tricuspid valve; other abbreviations as in Figures 1 and 2.

The patient maintained oxygen saturation levels >90%, with adequate spontaneous diuresis, enteral feeding, and progressive weight gain. Serial blood tests showed a marked reduction in cardiac neurohormones, indicating improved cardiac function (Figures 2 and 3). Consequently, the patient was discharged.

## Outcome and Follow-Up

**Figure 4:**
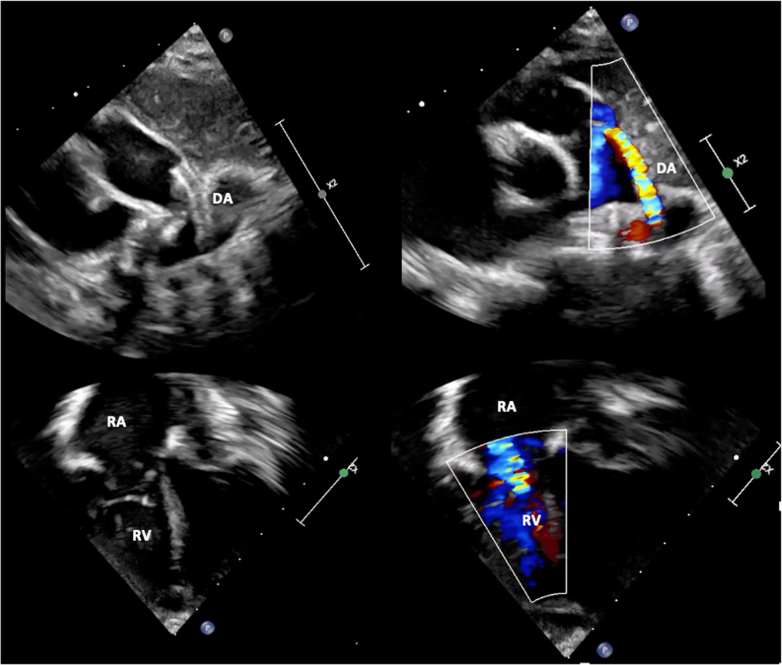
Follow-Up Transthoracic Echocardiography at Age 18 Months (Top Panels) Parasternal views showing the stent of the ductus arteriosus, with sustained retrograde flow into the pulmonary arteries. (Bottom Panels) Apical 4-chamber views demonstrating right ventricular growth and reduced tricuspid valve regurgitation, supporting successful right ventricular remodeling and progressive transition toward biventricular circulation. Abbreviations as in Figure 1.

The child underwent regular follow-up appointments every 3 weeks. At home, he remained in good condition and continued to experience weight gain. At the age of 15 months, follow-up echocardiography revealed an increase in end-diastolic RV volume, with preserved contractility despite moderate diastolic dysfunction. There was a significant increase in anterograde pulmonary blood flow, and the ductus stent remained patent, directing flow from the aorta to the pulmonary trunk with a maximum velocity of 3.5 m/s. Moderate tricuspid valve regurgitation and a transtricuspid mean gradient of 2 mm Hg were observed, while other cardiac structures remained unchanged (Figure 4).

## Discussion

TVD and PVD are rare congenital heart diseases.^1^ The association of these conditions is rarely described, and their combined management is even more challenging. During gestation, TVD posed the most significant concern owing to the risk of fetal hydrops. The absence of anterograde pulmonary flow could be attributed to atresia/severe pulmonary stenosis or functional atresia secondary to severe tricuspid regurgitation. The tricuspid valve annulus *z*-score and RV end-diastolic volume were borderline, making it difficult to decide between the univentricular versus biventricular strategies.^2^, ^3^, ^4^ Fetal cardiac ultrasound allowed physicians to monitor the evolution and plan future newborn care.^5^ Notably, at birth, tricuspid regurgitation was only moderate, whereas the predominant clinical feature was severe stenosis of the pulmonary valve and inadequate forward flow of the RV, akin to critical pulmonary stenosis with an intact ventricular septum.

The treatment goal was to achieve a biventricular circulation with recruitment of the RV. Early administration of PGE1 at birth facilitated the attainment of adequate pulmonary blood flow. Transcatheter pulmonary valve perforation effectively enhanced the RV outflow tract^6^ and reduced the gradient through the tricuspid, representing a viable option for initial palliation. However, the procedure alone proved insufficient, as the pulmonary circulation remained ductal dependent. Subsequently, DA stenting was performed to ensure adequate pulmonary blood flow. The pulmonary regurgitation resulting from valvuloplasty allowed for volumetric overload of the RV, fostering development and growth. Interestingly, the same tricuspid regurgitation that was a primary concern during pregnancy later contributed to RV development. Importantly, coronary angiography revealed no coronary sinusoids or RV-dependent coronary circulation, with significant prognostic implications.^7^

The initial choice of a percutaneous rather than surgical approach was the result of a multidisciplinary discussion involving the pediatric cardiology, cardiac surgery, and interventional cardiology teams. The patient's hemodynamic stability, preserved left ventricular function, and absence of RV-dependent coronary circulation supported a less invasive strategy, avoiding neonatal cardiopulmonary bypass and allowing staged assessment of RV growth. Possible limitations of the transcatheter strategy include the persistence of ductal dependence in some cases, the potential for reintervention, and the inability to correct valve dysplasia directly at the initial stage. Surgery was deferred with the expectation that definitive repair will be undertaken at a more favorable time.

This combined percutaneous strategy proved successful, allowing the RV and pulmonary branches to grow and ultimately enabling the choice of biventricular circulation versus univentricular or one-and-a-half ventricular repair. Several studies have proposed echocardiographic parameters to predict the feasibility of a biventricular heart strategy and identify cases of ductal dependence.^8^, ^9^, ^10^

Unfortunately, future cardiosurgical intervention may be required given the anatomical and functional abnormalities of the right-sided valves, which will be addressed surgically at the optimal time. Patients with congenital heart disease present unique challenges, and standardized treatment approaches may not be applicable. Each therapeutic intervention must be tailored to the individual patient, considering the underlying pathophysiological mechanisms and carefully pondering the costs and benefits of each intervention.

## Conclusions

Prenatal diagnosis of TVD with PVD allowed timely planning and early execution of biventricular repair, with promising results for long-term outcomes. A staged, individualized strategy can facilitate RV growth in this setting.

## Funding Support and Author Disclosures

Dr Olivotto has received advisory board fees/research grants from Bristol Myers Squibb, Cytokinetics, Amicus, Sanofi Genzyme, Bayer, Tenaya, Rocket Pharma, Lexeo, Chiesi, and Boston Scientific. All other authors have reported that they have no relationships relevant to the contents of this paper to disclose.
